# Cost-effectiveness and cost-utility of a therapist-guided online intervention provided soon after trauma: Results from a randomized controlled trial

**DOI:** 10.1016/j.invent.2025.100886

**Published:** 2025-11-06

**Authors:** Maria Bragesjö, Filip K. Arnberg, Erik Andersson

**Affiliations:** a Department of Clinical Neuroscience, Centre for Psychiatry Research, Karolinska Institutet, & Stockholm Health Care Services, Sweden; b National Centre for Disaster Psychiatry, Department of Neuroscience, Psychiatry, Sweden; c Stress Research Institute, Stockholm University, Sweden; d Department of Clinical Neuroscience, Division of Psychology, Karolinska Institutet, Sweden

**Keywords:** Posttraumatic stress disorder, Digital mental health, Early intervention, Trauma, Prevention, Cost-utility, Cost-effectiveness

## Abstract

This study evaluated the cost-effectiveness and cost-utility of a therapist-guided, internet-delivered early intervention for trauma. Exposure to traumatic events is common and can lead to substantial psychological distress, functional impairment, and societal costs. Early psychological interventions have the potential to mitigate these effects. We developed Condensed Internet-delivered Prolonged Exposure (CIPE), a digital intervention delivered within two months of trauma exposure. In a randomized controlled trial (*N* = 102), CIPE was more effective than a waiting-list control in reducing post-traumatic stress symptoms at post-intervention (3 weeks) and at a prespecified 7-week follow-up while the waiting-list control remained intact (prior to crossover). In this study, we evaluated CIPE from a societal cost perspective, aggregating direct medical costs (healthcare contacts, medication) and indirect costs (sick leave, reduced productivity, domestic loss) with equal weight in total cost calculations using a self-report questionnaire. Cost-effectiveness was assessed using responder status (≥10-point symptom reduction) and subthreshold symptom status on the PTSD Checklist for DSM-5. Cost-utility was assessed using quality-adjusted life years (QALYs) from the EQ-5D. Incremental cost-effectiveness ratios (ICERs) were estimated using bootstrapped regression analyses and visualized in cost-effectiveness planes and acceptability curves. CIPE showed a 95 % probability of being cost-effective at a willingness-to-pay threshold of €939–1181 per additional responder or subthreshold case. The corresponding cost per QALY gained was €2929–3636. Effects were sustained at 12-month follow-up. These findings suggest that therapist-guided digital exposure therapy delivered soon after trauma can reduce symptoms at a relatively low cost to society. Future research should examine CIPE's long-term economic impact and potential for broader implementation.

## Highlights

• CIPE is a 3-week, therapist-guided digital intervention soon after trauma that reduced posttraumatic stress symptoms at 3- and 7-week follow-ups
• CIPE showed a 95% probability of being cost-effective versus a waiting-list control.
• One QALY gained with CIPE costs €2929 to €3636 from a societal perspective.
• Effects were sustained at 12 months, supporting early integration into mental health care.

## 1 Introduction

As much as 70 % of people will experience a potentially traumatic event in their lifetime and some of these individuals also develop more severe psychiatric symptoms such as acute stress disorder or post-traumatic stress disorder (PTSD) (Kessler et al., 2017). PTSD is associated with a substantial societal burden, including elevated levels of sick leave (Alonso et al., 2011; Kessler, 2000; Wald and Taylor, 2009; Lim et al., 2000), unemployment (Wald and Taylor, 2009; Kessler and Frank, 1997; Davis et al., 2022), homelessness (Wald and Taylor, 2009; Davis et al., 2022; Surís et al., 2004), educational impairments (Vilaplana-Pérez et al., 2020), increased use of healthcare and social services (Greenberg et al., 1999; Kessler et al., 1995), and impaired work performance (Wald and Taylor, 2009). Face-to-face, trauma-focused cognitive-behavioral therapy (CBT) is an effective first-line treatment for PTSD (Lewis et al., 2020).

One way to further innovate current treatments for trauma-related problems could be to deliver scalable psychological interventions shortly after a traumatic event instead of waiting until it has developed into a psychiatric disorder such as PTSD. This approach could not only be potentially cost-effective but also be essential for reducing the overall burden of PTSD on society. Our research group has developed and evaluated a digital intervention, Condensed Internet-delivered Prolonged Exposure (CIPE) in one pilot study and one large-scale efficacy trial. Administered within the first two months after trauma exposure, CIPE was found to be feasible, acceptable, and effective in reducing post-traumatic stress symptoms up to a six-month follow-up (Bragesjö et al., 2021a; Bragesjö et al., 2021b). Trauma-focused CBT for PTSD has been estimated to cost approximately $36,893 per additional remission (Slade et al., 2017), and $19,000 per QALY (Mihalopoulos et al., 2015) which highlights the need to investigate whether more scalable and accessible interventions can achieve similar effects at lower costs. While treatment completion has been associated with reduced service utilization (Tuerk et al., 2013), capacity constraints and access barriers limit scalability. We therefore evaluated the cost-effectiveness and cost-utility of CIPE from a societal perspective, quantifying the incremental cost per additional responder and per subthreshold case, as well as cost per QALY gained. Further, we also report previously unpublished 12-month follow-up data on the clinical outcomes for CIPE.

## 2 Method

### 2.1 Design and setting

**Fig. 1:**
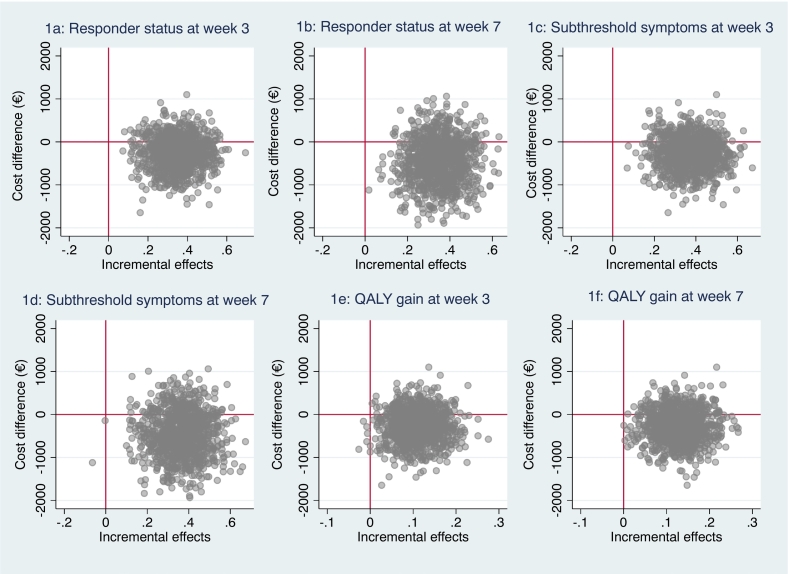
a–f: Cost-effectiveness planes.

We used health economic data from a randomized controlled trial in which 102 individuals who had recently experienced a psychological trauma were allocated to either the online intervention, CIPE, or to a waiting-list control group (Bragesjö et al., 2021a). Assessment points were pre- (week 0) and post-intervention (week 3) as well as a controlled 1-month follow-up (week 7). After the 1-month follow-up, the control group was crossed over to CIPE. Naturalistic long-term follow-ups were subsequently conducted at 6- and 12-months for the CIPE group. The study design with assessment points is visualized in Fig. 1. Data were collected between October 2019 and June 2020. The trial was approved by the National Ethical Review Board in Sweden (registration ID: 2019-04413), and pre-registered at ClinicalTrials.gov (registration ID: NCT03850639).

### 2.2 Recruitment and participants

The trial employed online self-referral as recruitment strategy and was advertised in social media and nationwide newspapers. Eligibility criteria were assessed by a psychologist through structured telephone interviews with potential participants. The target population was Swedish residents exposed to a traumatic event according to criterion A for PTSD in the fifth version of the Diagnostic and Statistical Manual of Mental Disorders (DSM-5) within two months prior to inclusion (i.e., exposed to actual or threatened death, serious injury, or sexual violence (American Psychiatric Association, 2013)) with at least a total score of ≥10 points on the PTSD Symptom Checklist for DSM-5 (PCL-5). Exclusion criteria were: a) other serious psychiatric comorbidity as the primary concern (e.g., on-going substance abuse, untreated bipolar disorder, psychotic symptoms, severe depression, or high suicide risk); b) currently receiving CBT for trauma-related distress; and c) ongoing trauma-related threat (e.g. living with a violent spouse). Participants using psychotropic medication had to report a stable dose for two weeks prior to inclusion in the study. Table 1 briefly reports patients' demographics at baseline. The sample primarily comprised college-educated women in their early forties who during the past month had been exposed to a potentially traumatic event. The three most common trauma types were exposure to interpersonal violence (28 %), death (25 %) and rape (16 %). Approximately one-third of the sample participants reported that they currently were on sick leave. Please see the original publication (Bragesjö et al., 2021a) for further details.

**Table 1:** Sample demographics.

Baseline characteristics		CIPE ^(N=51)^	WL ^(*N*=51)^
Gender, *n (%)*	Women	45 (88 %)	39 (76 %)
Age	Mean *(SD)*	44.7 (17)	36.7 (13.4)
	Range	18–82	18–75
Highest education, *n (%)*	College/university	30 (57 %)	25 (47 %)
Occupational status, *n (%)*	Working full time	15 (29 %)	20 (39 %)
	On sick leave	17 (33 %)	15 (29 %)
Type of trauma, *n (%)*	Rape/interpersonal violence	27 (53 %)	28 (55 %)
	Non-intentional	24 (47 %)	23 (45 %)
Days since exposure to index trauma, mean (SD)	Mean (*SD*)	36.3 (19)	35.4 (17.11)
	Range	6–65	4–62

Abbreviations: CIPE, Condensed Internet-delivered Prolonged Exposure; WL; waiting list.

### 2.3 Intervention

The experimental group was provided a 3-week therapist-assisted digital intervention that comprises about 30 pages of written self-help material. The treatment rationale was inspired by a condensed version of prolonged exposure adapted to be delivered in the early aftermath of a traumatic event (Rothbaum et al., 2012). Module 1 contained psychoeducation about common reactions to trauma and a general treatment rationale. In module 2, the participants were encouraged to engage in daily imaginal exposure (revisit and recount the memory of the traumatic event) for 20–30 min followed by 15 min of cognitive processing to allow enough time to reflect upon new ways to think about the trauma. In module 3, the participants were encouraged to continue with imaginal exposure and approach the most distressing parts of their traumatic memory and to gradually break behavioral avoidance. In the last module, the participants were asked to summarize their progress and learning and make a relapse prevention plan.

The treatment program used five case examples of recently traumatized individuals representing both genders and both interpersonal and non-interpersonal events. The case examples depicted common difficulties encountered during exposure and were followed throughout all modules, illustrating progress through each intervention component. Digital worksheets were used where the participant could track their progress and observe changes in emotional intensity and negative beliefs about themselves, others, or the world. The therapists guided the participants through the intervention and provided feedback on completed assignments, answered questions, and provided support. Reminders were sent out in the form of text-messages and phone calls if the participant did not log in the digital platform for three days. The total mean therapist time per participant during the intervention period of 3 weeks in the CIPE group was 46 min (SD = 48).

Participants who were randomized to the control group were informed that they could contact study personnel if their symptoms worsened. We also made sure that they knew how to come in contact with regular care if needed. The main idea with this type of control group was to provide what most individuals receive after exposure to a potentially traumatic event and to control for natural recovery.

### 2.4 Cost-data assessments

Health economic cost data were obtained at pre-intervention (week 0), post-intervention (week 3) and at the controlled 1-month follow-up (week 7) using an adapted version of the Trimbos and Institute of Medical Technology Assessment Cost Questionnaire for Psychiatry (TIC-P; (Hakkaart-van Roijen and Donker, 2002)). This self-rating questionnaire collects data on health care visits (e.g. “how many visits to [profession] have you done the last 2 weeks?”, medication consumption (e.g. “what kind of prescribed medication have you been using the last 2 weeks? Please write label, dosage and how many pills for each day”, unemployment (“are you currently unemployed?”), sick leave (how many days the last 2 weeks have you stayed home from work due to sick leave?”), as well as work cutback (e.g. “how many days the last 2 weeks have you been working with reduced capacity and at what level?”) and household cutback (e.g. “how many days the last 2 weeks have you not been able to do household work?”). The TIC-P thus enabled assessment of both direct and indirect costs. As the trial was conducted partly during the Covid-19 pandemic, we excluded costs related to unemployment from the TIC-P as this type of cost may have been heavily influenced by the pandemic. All costs were estimated using the national tariffs in Sweden.

The costs were originally computed in Swedish Crowns and then converted into Euros (€; exchange rate set on January 2022). We used the human capital approach which means that costs related to work loss were estimated based on the average gross earnings in Sweden for 2022 (Husereau et al., 2022). Domestic loss hourly tariff was estimated to €13 based on a previous study using the TIC-P (Smit et al., 2006). We used the full economic cost of a licensed clinical psychologist (€193 per visit) and multiplied this figure by the total time the therapist spent on each participant. Costs of the online platform for screening and assessment, or the cost of the automated reminders to complete the outcome assessments were not included as this was identical in both groups.

### 2.5 Assessment of clinical outcomes

For the primary cost-effectiveness analyses, we used responder status (yes/no) defined as ≥10 points reduction from baseline to post- or follow-up on the PTSD Checklist for DSM-5 (PCL-5) (Weathers et al., 2013). We also used subthreshold symptom status which was defined as <30 points on the PCL-5 at post- or follow-up based on a study conducted in a Swedish sample (Bondjers, 2020).

For the cost-utility analysis, we used the EuroQol Questionnaire-5 dimensions (EQ-5D; (Rabin and de Charro, 2001) which is a generic, health-related quality of life to estimate population-based health utilities. The scoring ranges from 0 (dead) to 1 (perfect health). In the present study, EQ-5D was used to assess quality adjusted life years (QALYs) where the standard interpretation is that 1 QALY stands for one year of perfect health.

### 2.6 Statistical analyses

Economic data obtained from the TIC-P was first calculated and estimated for each assessment point (pre-intervention [week 0], post-intervention [week 3] and 1-month follow-up [week 7]). We used a societal perspective which included all direct and indirect costs. This type of analysis provides an estimation of how cost-effective CIPE is from multiple payers, e.g. the health care system, the government, the employer. We then tested if there were any between-group changes in costs from pre-intervention to post-intervention and follow-up in any of the cost-related domains (i.e. health care visits, medications, direct non-medical costs, sick leave, work cutback and/or domestic loss). This was conducted using a linear regression framework with robust standard errors and bootstrap replications (*n* = 1000), with the 3- and 7-week costs as dependent variable and group and baseline costs as independent variables. Thirdly, incremental cost-effectiveness ratios (ICER) were estimated by dividing the accumulated between-group costs at week 3 (baseline costs + costs week 3 + intervention costs) and week 7 (baseline costs + costs week 3 + costs week 7 + intervention costs) with the incremental between-group effectiveness; in this case responder status, subthreshold symptom status and incremental gains on the EQ-5D (i.e. [Δ^C1^ – Δ^C2^]/[Δ^E1^ – Δ^E2^]). This was also estimated using non-parametric bootstrapping regression estimations that were plotted in cost-effectiveness planes. We also calculated cost-effectiveness acceptability curves with different societal willingness-to-pay values for one unit of improvement (in this case one additional case of a responder or minimal symptoms, or a QALY) (Drummond et al., 2005).

To investigate long-term effects, we conducted a paired *t*-test from pre-intervention to the 12-month follow-up. We also estimated long-term economic impact using a bootstrapped regression framework with cost-change from pre-intervention to the 12-month follow-up as the dependent variable for the CIPE group only. All statistical analyses were conducted using STATA IC/16.1 (Statacorp, USA).

## 3 Results

### 3.1 Cost change and clinical outcomes

Mean costs that were estimated using the TIC-P are presented in eTable 1. eTable 2 shows detailed statistics from the estimated between-group cost-change. Notably, the CIPE group had overall greater reductions in costs from baseline during the intervention period than the control group. The number of responders in the CIPE group was 26/44 and 12/50 in the control group at post-intervention (week 3). The number of participants who experienced subthreshold symptoms (<30 points on the PCL-5) was 27 of 44 in the CIPE group and 12 of 50 in the control group at this time point. The corresponding figure at the 1-month follow-up (week 7) was 31 of 43 responders in the CIPE group and 18 of 48 in the control group. At week 7, 30 of 43 individuals in the CIPE group and 16 of 48 individuals the control group were classified as belonging to the subthreshold symptoms group. The mean QALY gain on the EQ-5D for the CIPE group vs. the waiting-list control group was 0.11 (95 % CI 0.02–0.20) from week 0 to week 3 and 0.13 (95 % CI 0.04–0.22) from week 0 to week 7 (means for the EQ-5D can be found in the main outcome publication; 16).

### 3.2 Cost-effectiveness analysis

**Fig. 2:**
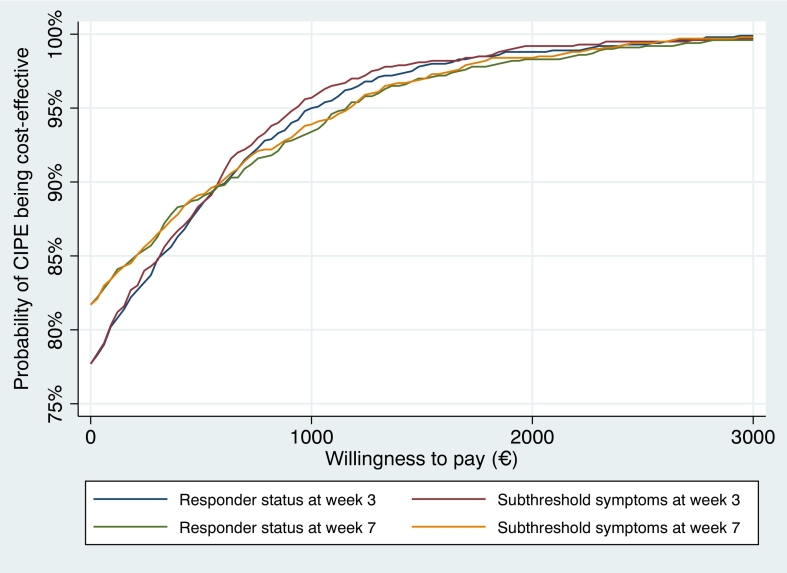
Cost-effectiveness acceptability curves on clinical efficacy.

The mean ICER for the responder analysis at week 3 was −271 (bootstrapped cost change) / 0.35 (bootstrapped clinical efficacy coefficients) = −774 which means that one additional responder was on average associated with a societal earning of €774. Fig. 1a displays the cost-effectiveness plane from this analysis and Fig. 2 shows the cost-effectiveness given different willingness-to-pay scenarios. The results from the latter analysis showed that CIPE had a 95 % probability of being a cost-effective intervention given a willingness-to-pay of €1000 for one additional responder. When using subthreshold symptoms as outcome, the corresponding ICER was estimated to −271 (bootstrapped cost change) / 0.38 (bootstrapped clinical efficacy in responder status) = −729 with the CIPE intervention standing 95 % of being a cost-effective assuming a willingness-to-pay of €939 for one additional case of being in the subthreshold symptoms group (Figs. 1b and 2).

The analyses were repeated but using results on week 7 as main outcome. The results showed that the estimated mean ICER of CIPE was −450 / 0.35 = −557 when using the responder and −450 / 0.37 = −1773 when using the subthreshold symptoms criterium. Fig. 1c and d shows the cost-effectiveness planes from these analyses and Fig. 2 shows the results based on different willingness-to-pay scenarios. Here, the CIPE intervention had 95 % probability of being cost-effective given a value of €1181 per responder as well as for an additional case of subthreshold symptoms.

### 3.3 Cost-utility analysis

**Fig. 3:**
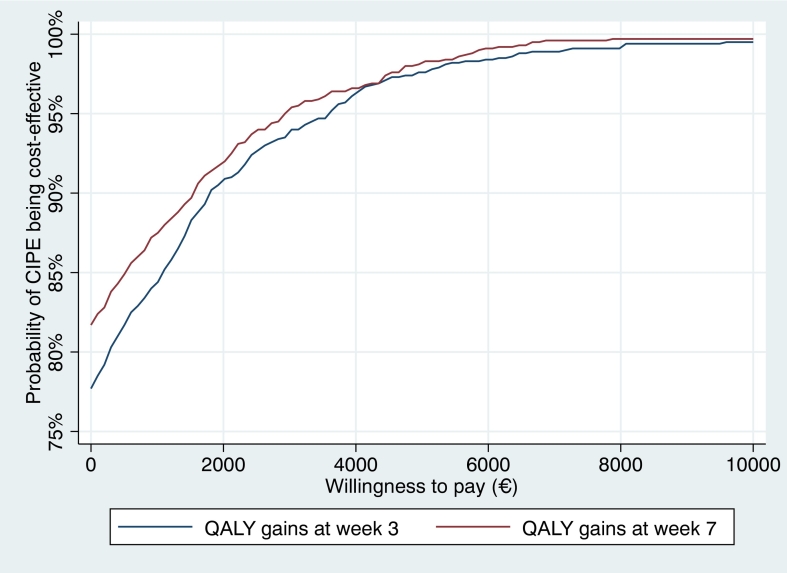
Cost-effectiveness acceptability curves on QALY gains.

When using the EQ5D as outcome, the mean ICER in the bootstrapped regression analysis was estimated to −271 / 0.11 = −4237 at week 3 (Fig. 1e) and −450 / 0.13 = −7698 at week 7 (Fig. 1f). Fig. 3 shows acceptability curves for these cost-utility analyses. Here, the results showed that CIPE had a probability of 95 % being cost-effective given a willingness-to-pay of €2929–3636.

### 3.4 Uncontrolled long-term follow-up data

The mean score on the PCL-5 at baseline for the CIPE group was 42.59 (SD = 14.25) and the corresponding figure at the 12-month follow-up was 15.10 (SD 13.60). A paired *t*-test indicated that participants who had undergone CIPE had significant reductions from pre-intervention to the 12-month follow up (t_37_ = 11.30, *p* < .001) with a within-group effect size estimated to *d* = 1.97 (95 % CI; 1.45–2.47) and 33 of 38 individuals classified as responders and had subthreshold symptoms (statistics on the secondary outcomes is provided in eTable 3). We also investigated differences in costs between pre-intervention and the 12-month follow-up for the CIPE group. Results showed that participants who had received CIPE had significantly lower costs at the 12-month follow-up related to indirect non-medical costs (B = −517, Z = −3.58, *p* < .001) which were mainly driven by reductions in sick leave (B = −445, Z = −3.29, *p* < .001).

## 4 Discussion

Previous research has shown that PTSD symptoms are common in the population and can lead to increased levels of sick leave, unemployment, homelessness, educational impairments as well as impaired performance at work (Alonso et al., 2011; Kessler, 2000; Wald and Taylor, 2009; Lim et al., 2000; Kessler and Frank, 1997; Davis et al., 2022; Surís et al., 2004; Vilaplana-Pérez et al., 2020). This highlights the need for using health economic analyses when developing and testing novel interventions for trauma-related problems. The current study is the first step in evaluating the cost-effectiveness of an early-provided online intervention (CIPE) for individuals who recently has experienced a traumatic event. In economic terms, CIPE compares favorably with established benchmarks for face-to-face trauma-focused CBT. Results showed that CIPE had a 95 % probability of being cost-effective given that society is willing to accept the cost of €939–€1181 for an additional case of a responder and/or a case with subthreshold trauma symptoms. Published estimates for CBT are approximately $36,893 per additional remission and $19,000 per QALY (Slade et al., 2017; Mihalopoulos et al., 2015) whereas our early intervention after trauma estimates for CIPE are approximately €939–1181 per additional responder/subthreshold case and €2929–3636 per QALY under a societal perspective. These values fall well below commonly cited decision thresholds (e.g., NICE) and indicate a substantial relative economic advantage, although we note that CIPE is delivered early post-trauma whereas published CBT estimates often reflect the cost of achieving remission in more chronic presentations (McCabe et al., 2008). The costs 12 months post intervention indicated further cost reductions and sustained long-term effects for individuals who have received the CIPE intervention, with 87 % of participants being responders and having subthreshold symptoms according to the PCL-5. One plausible explanation for the sustained effects observed at 12-month follow-up is consolidation of exposure learning. By repeatedly engaging in imaginal and in vivo exposure, participants may have reduced the perceived threat of trauma-related cues, thereby strengthening inhibitory learning processes and attenuating fear responses over time. A second mechanism concerns reductions in avoidance behaviors, which not only facilitate natural recovery but also prevent the reinforcement of maladaptive coping strategies. In fact, in a companion study using the same sample, we found that decreases in avoidance during the intervention period statistically mediated improvements across other PTSD symptom clusters at follow-up (Bragesjö et al., 2024). Finally, the relapse prevention component may have enhanced self-efficacy and maintenance strategies, encouraging participants to continue applying skills beyond the active treatment phase. Taken together, these mechanisms may help explain why a brief, early intervention such as CIPE can yield durable clinical and economic benefits.

From a policy perspective, our results offer early, practice-oriented cost-effectiveness evidence for therapist-guided digital exposure delivered soon after trauma. In light of commonly used decision thresholds and reporting guidance (e.g., CHEERS; NICE), the estimated cost per responder and per QALY suggests that CIPE merits consideration for large-scale implementation in digitally enabled mental health services.

Strengths with the current study were the randomized design, the relatively large sample size and controlled follow-ups at 1-month post-intervention. We also note some major limitations: First, we used self-rated questionnaires to assess costs and trauma symptoms which may have caused some bias in the estimates. Future studies could instead use register-based data to collect health-economic data and clinical outcomes could be assessed by blinded clinicians. A second limitation with the current study was that we did not incorporate treatment maintenance- and developmental costs for the online platform. Specific investment costs related to renting or developing a treatment platform combined with resources needed for organizing clinical routines and assessment procedures should therefore be considered when interpreting the results from the current study. A third limitation was that the long-term follow-ups were naturalistic due to the fact that the waiting-list was offered CIPE. It is thus possible that the improvements seen in sick-leave from pre-intervention to the 12-month follow-up were at least partly influenced by Covid-19 vaccine rollouts. One important next step for future research could therefore to be use controlled long-term follow-ups and to investigate if CIPE has a preventive effect against the build-up of full PTSD, and if this in turn can lead to a reduced societal burden.

Altogether, this study indicates that it is possible to alleviate early trauma symptoms in individuals to a relatively low societal cost. More research is needed to investigate if CIPE reduce the long-term societal economic burden associated with traumatic events.

## Ethical standards

All procedures were conducted in accordance with the Declaration of Helsinki. Ethical approval was obtained from the National Ethical Review Board in Sweden (registration ID: 2019-04413), and the trial was pre-registered at ClinicalTrials.gov (ID: NCT03850639). Written informed consent was obtained from all participants.

## Funding

This work was supported by the 10.13039/501100004359Swedish Research Council (EA, grant 2016-02359), the 10.13039/501100007687Swedish Society for Medicine (EA, grant 658811) and Stockholm County Healthcare (EA, grant 20170018). None of the funding organisations had any role in the conception of the study design or in the collection, analysis or interpretation of the data, in the writing of the report, or in the decision to submit the paper.

## Declaration of competing interest

No potential conflict of interest was reported by the authors.

## Data Availability

The raw data supporting the conclusions of this article will be made available by the authors upon request given that the request comply with Swedish and EU laws regulating protection of identifiable data.

## References

[bb0005] Alonso J., Petukhova M., Vilagut G., Chatterji S., Heeringa S., Üstün T.B. (2011). Days out of role due to common physical and mental conditions: results from the WHO World Mental Health surveys. Mol. Psychiatry.

[bb0010] American Psychiatric Association (2013).

[bb0015] Bondjers K. (2020).

[bb0020] Bragesjö M., Arnberg F.K., Olofsdotter Lauri K., Aspvall K., Särnholm J., Andersson E. (2021). Condensed Internet-delivered prolonged exposure provided soon after trauma: a randomised trial. Psychol. Med..

[bb0025] Bragesjö M., Arnberg F.K., Särnholm J., Olofsdotter Lauri K., Andersson E. (2021). Condensed internet-delivered prolonged exposure provided soon after trauma: a randomised pilot trial. Internet Interv..

[bb0030] Bragesjö M., Arnberg F.K., Andersson E. (2024). Mediators of change in a condensed online exposure-based intervention provided soon after trauma: insights from a randomised controlled trial. Eur. J. Psychotraumatol..

[bb0035] Davis L.L., Schein J., Cloutier M., Gagnon-Sanschagrin P., Maitland J., Urganus A. (2022). The economic burden of posttraumatic stress disorder in the United States from a societal perspective. J. Clin. Psychiatry.

[bb0040] Drummond M.F., Sculpher M.J., Torrence G.W., O’Brien B.J., Stoddart G.L. (2005).

[bb0045] Greenberg P.E., Sisitsky T., Kessler R.C., Finkelstein S.N., Berndt E.R., Davidson J.R. (1999). The economic burden of anxiety disorders in the 1990s. J. Clin. Psychiatry.

[bb0050] Hakkaart-van Roijen L., Donker M.C.H. (2002). Manual/iMTA Questionnaire for Costs Associated with Psychiatric Illness (TIC-P). http://hdl.handle.net/1765/1337.

[bb0055] Husereau D., Drummond M., Augustovski F., de Bekker-Grob E., Briggs A.H., Carswell C. (2022). Consolidated Health Economic Evaluation Reporting Standards 2022 (CHEERS 2022) statement: updated reporting guidance for health economic evaluations. BMC Med..

[bb0060] Kessler R.C. (2000). Posttraumatic stress disorder: the burden to the individual and to society. J. Clin. Psychiatry.

[bb0065] Kessler R.C., Frank R.G. (1997). The impact of psychiatric disorders on work loss days. Psychol. Med..

[bb0070] Kessler R.C., Sonnega A., Bromet E., Hughes M., Nelson C.B. (1995). Posttraumatic stress disorder in the national comorbidity survey. Arch. Gen. Psychiatry.

[bb0075] Kessler R.C., Aguilar-Gaxiola S., Alonso J., Benjet C., Bromet E.J., Cardoso G. (2017). Trauma and PTSD in the WHO world mental health surveys. Eur. J. Psychotraumatol..

[bb0080] Lewis C., Roberts N.P., Andrew M., Starling E., Bisson J.I. (2020). Psychological therapies for post-traumatic stress disorder in adults: systematic review and meta-analysis. Eur. J. Psychotraumatol..

[bb0085] Lim D., Sanderson K., Andrews G. (2000). Lost productivity among full-time workers with mental disorders. J. Ment. Health Policy Econ..

[bb0090] McCabe C., Claxton K., Culyer A.J. (2008). The NICE cost-effectiveness threshold: what it is and what that means. Pharmacoeconomics.

[bb0095] Mihalopoulos C., Magnus A., Lal A., Dell L., Forbes D., Phelps A. (2015). Is implementation of the 2013 Australian treatment guidelines for posttraumatic stress disorder cost-effective compared to current practice? A cost-utility analysis using QALYs and DALYs. Aust. N. Z. J. Psychiatry.

[bb0100] Rabin R., de Charro F. (2001). EQ-5D: a measure of health status from the EuroQol Group. Ann. Med..

[bb0105] Rothbaum B.O., Kearns M.C., Price M., Malcoun E., Davis M., Ressler K.J. (2012). Early intervention may prevent the development of posttraumatic stress disorder: a randomized pilot civilian study with modified prolonged exposure. Biol. Psychiatry.

[bb0110] Slade E.P., Gottlieb J.D., Lu W., Yanos P.T., Rosenberg S., Silverstein S.M. (2017). Cost-effectiveness of a PTSD intervention tailored for individuals with severe mental illness. Psychiatr. Serv..

[bb0115] Smit F., Willemse G., Koopmanschap M., Onrust S., Cuijpers P., Beekman A. (2006). Cost-effectiveness of preventing depression in primary care patients: randomised trial. Br. J. Psychiatry.

[bb0120] Surís A., Lind L., Kashner T.M., Borman P.D., Petty F. (2004). Sexual assault in women veterans: an examination of PTSD risk, health care utilization, and cost of care. Psychosom. Med..

[bb0125] Tuerk P.W., Wangelin B., Rauch S.A., Dismuke C.E., Yoder M., Myrick H. (2013). Health service utilization before and after evidence-based treatment for PTSD. Psychol. Serv..

[bb0130] Vilaplana-Pérez A., Sidorchuk A., Pérez-Vigil A., Brander G., Isoumura K., Hesselmark E. (2020). Assessment of posttraumatic stress disorder and educational achievement in Sweden. JAMA Netw. Open.

[bb0135] Wald J., Taylor S. (2009). Work impairment and disability in posttraumatic stress disorder: a review and recommendations for psychological injury research and practice. Psychol. Inj. Law.

[bb0140] Weathers F.W., Litz B.T., Keane T.M., Palmieri P.A., Marx B.P., Schnurr P.P. (2013). Scale Available from the National Center for PTSD at www ptsd va gov.

